# Preliminary investigation of anti-fatigue effects and potential mechanisms of meiju oral liquid in mouse and zebrafish models

**DOI:** 10.1371/journal.pone.0316761

**Published:** 2025-03-06

**Authors:** Lanlan Zhang, Jingcheng Zhao, Xi Zhou, Maitinuer Maiwulanjiang

**Affiliations:** 1 State Key Laboratory Basis of Xinjiang Indigenous Medicinal Plants Resource Utilization, Xinjiang Technical Institute of Physics and Chemistry, Chinese Academy of Sciences, Urumqi, People’s Republic of China; 2 University of Chinese Academy of Sciences, Beijing, People’s Republic of China; 3 Xinjiang Production and Construction Corps Food and Drug Evaluation and Verification Center, Urumqi 830002, China; 4 Uyghur Medical Hospital of Xinjiang Uyghur Autonomous Region, Urumqi, People’s Republic of China; 5 Guangdong Provincial Key Laboratory of Chemical Measurement and Emergency Test Technology, Guangdong Provincial Engineering Research Center for Quality and Safety of Traditional Chinese Medicine, Institute of Analysis, Guangdong Academy of Sciences, China National Analytical Center, Guangzhou, People’s Republic of China; Foshan University, CHINA

## Abstract

Meiju Oral Liquid (MOL), a representative medicinal formula in China, stems from the traditional use of specific Chinese medicinal herbs known for their anti-fatigue properties, including rose, jujube, chicory, and wolfberry. While these individual herbs have been recognized for their benefits, the formulation of MOL itself has not been extensively studied. This study was designed to evaluate the potential anti-fatigue effects of MOL, prepared from these natural herbs, and to explore its underlying mechanisms. In this research, both mouse and zebrafish models were utilized to investigate the anti-fatigue effects of MOL. Chemical characterization of MOL and identification of bioactive compounds in serum were conducted using ultra-performance liquid chromatography-quadrupole time-of-flight mass spectrometry (UPLC-Q-TOF/MS). The results demonstrated that MOL significantly prolonged the weight-bearing swimming time in mice, increased hepatic and muscle glycogen content, and reduced serum levels of blood urea nitrogen, blood lactate, and inflammatory markers (IL-1β, IL-6, TNF-α, and NO). Furthermore, MOL down-regulated the expression of NOX4 and TNF-α proteins while up-regulating p-PI3K and p-AKT proteins in the liver tissues of fatigued mice. In zebrafish models, MOL exhibited protective effects against sodium sulfite-induced lethality, enhanced high-speed motion trajectories, and increased movement distances in both normal and fatigued zebrafish. Additionally, MOL downregulated IL-1β, IL-6, TNF-α, and TNF-β mRNA levels while up-regulating PI3K and AKT1 mRNA levels in fatigued zebrafish. These findings suggested that the anti-fatigue effects of MOL may be mediated through the activation of the PI3K/AKT signaling pathway as well as the inhibition of TNF-α and NOX4 expression. In addition, a total of ninety-four chemical components were identified in MOL, with twenty-three migration compounds detected in mouse serum. These migration compounds are likely the primary active agents, contributing to the reduction of metabolite accumulation, enhancement of glycogen synthesis, and suppression of inflammatory responses. Taken together, our findings underscore the potential anti-fatigue effects of MOL, warranting further investigation into its therapeutic applications and the specific roles of its bioactive compounds.

## Introduction

Fatigue is a prevalent and multifaceted condition that significantly affects individuals’ quality of life and encompasses a range of physiological and biochemical disruptions, including energy depletion, lactic acid accumulation, metabolic product deposition, and cellular oxidative damage [1,2]. These disruptions can lead to symptoms such as insomnia, weakened immunity, and other health issues [3]. If not properly managed, fatigue can escalate into more severe conditions such as chronic fatigue syndrome, and over-training syndrome, and even contribute to the development of endocrine disorders and other organic diseases. Despite its widespread prevalence, current intervention strategies, such as energy supplementation, often fail to address the underlying causes of fatigue, limiting their long-term efficacy.

To overcome these limitations, there is a growing interest in developing new anti-fatigue treatments, particularly those based on traditional Chinese medicine (TCM). TCM formulations are valued for their natural safety, multifunctional properties, and ability to synergistically target multiple pathways associated with fatigue [4–6]. Among these, Meiju Oral Liquid (MOL) is a novel formulation comprising *Rosa rugosa* Thunb. (rose), *Cichorium intybus* L. (chicory), *Ziziphus jujuba* Mill. (jujube), and *Lycium chinense* Mill. (wolfberry), herbs traditionally recognized for their anti-fatigue effects [7–10]. MOL integrates these herbs, potentially offering a more comprehensive approach to fatigue management by addressing both metabolic and inflammatory pathways.

Previous studies from our research team have shown that MOL has a significant anti-fatigue effect, evidenced by its ability to prolong the weight-bearing swimming time [11] and rod rotation time in mice [12]. However, the molecular mechanisms and bioactive constituents responsible for these effects are not yet fully understood. Fatigue is often associated with inflammatory responses and oxidative stress, particularly through the release of pro-inflammatory cytokines like TNF-α and IL-6, which can trigger and sustain fatigue symptoms [13,14]. The PI3K/AKT signaling pathway plays a critical role in cell survival, metabolism, and inflammation regulation, making it a key target in understanding the anti-fatigue effects of MOL [15]. Additionally, NOX4, a member of the NADPH oxidase family, is a significant source of reactive oxygen species (ROS) and is implicated in oxidative stress-related damage, which is closely linked to fatigue development [16].

In this study, we aimed to elucidate the anti-fatigue effects and mechanisms of MOL using both fatigued mouse models and juvenile zebrafish models. Specifically, we explored the potential involvement of PI3K/AKT signaling pathway and the regulation of oxidative stress indicators such as NOX4 in MOL’s anti-fatigue action. Additionally, we employed ultra-performance liquid chromatography-quadrupole time-of-flight mass spectrometry (UPLC-Q-TOF/MS) to characterize the chemical components in MOL and identify the bioactive compounds absorbed into serum following oral administration. To our knowledge, this is the first study that not only investigates the anti-fatigue effects of MOL but also seeks to uncover the mechanisms underlying its action.

## Materials and methods

### Reagents and Materials

The urea nitrogen content assay kit (#BC1535), lactic acid content assay kit (#BC2235), and NO content assay kit (#BC1475) were provided by Beijing Solarbio Science & Technology Co., Ltd. Liver glycogen (#20200513) and muscle glycogen kits (#20200316) were obtained from Nanjing Jiancheng Bioengineering Research Institute. ELISA kits for IL-1β (#E-EL-M0037), IL-6 (#E-EL-M0044), and TNF-α (#E-EL-M3063) were supplied by Elabscience Biotechnology Co., Ltd (Wuhan).

Primary antibodies used in the study included NOX4 (#ab112414, Abcam), TNF-α (#ab6671, Abcam), PI3K(#ab302958), Phospho-PI3K (#ab278545, Abcam), AKT (#9272, CST), Phospho-AKT (#4058, CST), GAPDH (#TA-08, ZSGB-Bio).

The chemical reagents KCl (CAS#7447-40-7), NaCl (CAS#7647-14-5), CaCl_2_ (CAS#10043-52-4), and MgSO_4_ (CAS#7487-88-9) were sourced from Sinopharm Chemical Reagent Co., Ltd.

The analytical standards used in the study are listed in Table 1.

**Table 1.** Information on chemical standards.

Compound	CAS Number	Supplier
L-Phenylalanine	63-91-2	Tanmo Quality Inspection-Standard Material Center
p-coumaric acid	501-98-4	Shanghai Tongtian Biotechnology Co., Ltd
Magnoflorine	2141-09-5	Chengdu Ruifensi Biotechnology Co., Ltd
Isochlorogenic acid C	32451-88-0	Chengdu Ruifensi Biotechnology Co., Ltd
Isochlorogenic acid B	14534-61-3	Chengdu Ruifensi Biotechnology Co., Ltd
Hyperoside	482-36-0	Shanghai STende Standard Technical Service Co., Ltd
Chicoric acid	70831-56-0	Shanghai STende Standard Technical Service Co., Ltd
Ellagic acid	476-66-4	Shanghai STende Standard Technical Service Co., Ltd
Neochlorogenic acid	906-33-2	Shanghai STende Standard Technical Service Co., Ltd
Isochlorogenic acid A	2450-53-5	Shanghai STende Standard Technical Service Co., Ltd
Cryptochlorogenic acid	905-99-7	Shanghai STende Standard Technical Service Co., Ltd
Protocatechuic acid	99-50-3	Shanghai STende Standard Technical Service Co., Ltd
Rutin	100080-202012	National Institutes for Food and Drug Control
Chlorogenic acid	110753-202119	National Institutes for Food and Drug Control
Aesculetin	110741-202310	National Institutes for Food and Drug Control
Adenosine	110879-202204	National Institutes for Food and Drug Control
Luteolin-7-O-β-D-glucuronide	111968-201602	National Institutes for Food and Drug Control
Isoquercitrin	111809-202205	National Institutes for Food and Drug Control
Acanthoside B	7374-79-0	Guangzhou Jiatu Technology Co., Ltd
Gallic acid	149-91-7	Guangzhou Jiatu Technology Co., Ltd

### Animals

Fifty SPF-grade male KM mice (18 ~ 22 g) were obtained from the Animal Experimentation Center of Xinjiang Medical University (License No. SCXK (Xin) 2018-0003) to investigate the anti-fatigue effects of MOL and its underlying mechanisms. This study was approved by the Experimental Animal Ethics Committee of the Institute of Materia Medica, Xinjiang Uygur Autonomous Region (Ethics Approval No. XJIMM-20220110-1).

Twelve SPF-grade male KM mice (25–28 g) were procured from Zhu Hai Bestong (License No. SYXK (Yue) 2020-0229) for the purpose of conducting serum pharmacochemistry studies. The study protocol received approval from the Experimental Animal Ethics Committee of China National Analytical Center, Guangzhou (Ethics Approval No. W220018). All animal experiments were performed in strict accordance with the regulatory guidelines for the care and use of experimental animals as stipulated by the Guangdong Province administration.

For the zebrafish studies, AB strain wild-zebrafish were provided by the Zebrafish Drug Screening Platform, Institute of Biology, Shandong Academy of Sciences to investigate the anti-fatigue effects of MOL and the associated mechanisms.

Throughout the experimental procedures, mice were anesthetized with 2% isoflurane to mitigate stress and discomfort. At the conclusion of the study, euthanasia was performed via cervical dislocation under deep isoflurane anesthesia, ensuring that the animals did not experience pain. To further minimize suffering, all animals were provided with comprehensive care and were closely monitored for any signs of distress, with immediate intervention when necessary.

### Preparation of MOL

MOL was provided by the Research Center of Key Technology and Technological Engineering of Ethnic Medicine, Xinjiang Institute of Physical and Chemical Technology, Chinese Academy of Sciences. All medicinal materials of MOL were identified by Professor Lu Chunfang, Associate Researcher at the Xinjiang Technical Institute of Physics and Chemistry, Chinese Academy of Sciences.

To prepare MOL, 6 kg rose, 8 kg chicory, 10 kg jujube, 12 kg wolfberry were mixed, rinsed with water twice, and soaked for 1 h. The mixture was then extracted twice: first with 15 times the volume of water for 1.5 h, followed by 10 times the volume of water another for 1.5 h. The extracts were combined and concentrated to a final volume equivalent to a 1:5 ratio of raw materials to water.

To clarify the extract, ZTC1 + 1-III clarifier was applied. Clarifying agent B (10% of the concentrated solution) was added at 70 °C until flocculent precipitates formed. The solution was left to stand for 2 h, followed by the addition of clarifying agent A (half the volume of clarifying agent B). After sitting for 30 min at room temperature, the solution was left to separate for 12 h. The supernatant was collected, centrifuged at 5000 rpm for 10 min to remove impurities, bottled, and sterilized using the Pasteurization method at 105°C/30 min to obtain the MOL solution (0.18 g of raw drug per 1 mL).

### Evaluation of anti-fatigue effects of MOL using exercise-induced Fatigue mice model

#### Experimental grouping and model construction.

Male KM mice (n =  10 per group) were randomly assigned to five groups: Control, Model, and MOL-treated groups with different doses of 1.67 mL/kg (MOL-L), 3.34 mL/kg (MOL-M), and 6.68 mL/kg (MOL-H). All groups received treatment once daily for 14 consecutive days (Table 2). The drug doses for MOL were selected based on preliminary studies and the traditional usage of the herbal components. The doses ranged from a lower limit of 1.67 mL/kg, approximating the therapeutic dose, to a higher dose of 6.68 mL/kg to explore potential dose-dependent effects while ensuring safety.

**Table 2:** Experimental grouping and administration.

Group	Does	Clinical volume	Drug Delivery Volume	Number of doses	Dosing concentration
	mL/kg	multiplier	mL/kg/dose	Dose/day	
Blank	/	/	20	1	water
Model	/	/	20	1	water
MOL-L	1.67	1	20	1	1.67 mL diluted to 20 mL
MOL-M	3.34	2	20	1	3.34 mL diluted to 20 mL
MOL-H	6.68	4	20	1	6.68 mL diluted to 20 mL

The body weight of the mice in each group was recorded at the beginning of the experiment, every three days during the treatment period, and at the end of the experiment. This was done to monitor any changes in body weight that could indicate the overall health status of the mice and to assess the potential impact of MOL on body weight throughout the study.

To induce exercise fatigue, mice in all groups except the Control were subjected to a swimming exercise performance test at 1 h after the final administration. Mice were loaded with a constant weight corresponding to 7% of their body weight and individually placed in a columnar swimming test box filled with water at a depth of approximately 25 cm, maintained at 30 ±  2°C. The endpoint for the test was defined as the point when a mouse’s head remained submerged for more than 6 seconds, indicating physical exhaustion. The time to exhaustion (weight-bearing swimming time) was recorded for each mouse.

#### Measurement of plasma inflammatory factors by ELISA.

Following the completion of the weight-bearing swimming test, mice were allowed to rest for 30 min. Blood samples were then collected via enucleation, and serum was separated by centrifugation at 2500 rpm for 10 min at 4 °C. The levels of IL-1β, IL-6 and TNF-α as well as NO concentration were quantified using corresponding ELISA kits according to the manufacturer’s instructions.

#### Organ collection and index calculation.

After blood collection, the animals were euthanized by cervical dislocation under deep anesthesia. The heart, liver, spleen, lungs, and kidneys were carefully excised, rinsed with ice-cold saline, dried with filter paper, and weighed immediately. The organ index was calculated using the following formula: Organ index =  Organ weight (mg)/Body weight (g) ×  100.

#### Determination of liver and muscle glycogen content.

After the liver was excised and weighed, approximately 0.5 g tissue was excised and homogenized in ice-cold saline to produce 10% tissue homogenates. The homogenates were centrifuged at 3500 rpm for 10 min at 4 °C. Similarly, 0.5 g of gastrocnemius muscle from the left hind limb was excised, rinsed with normal saline, blotted dry, and homogenized in ice-cold saline to prepare 10% homogenates. The homogenates were also centrifuged at 3500 rpm for 10 min at 4 °C. The supernatants were collected and stored at -80 °C until analysis. Glycogen levels in the liver and muscle were determined using corresponding assay kits.

#### Western blot analysis of NOX4, TNF-α, and PI3K/akt signaling-related proteins in mouse liver tissue.

Mouse liver tissues were lysed on ice using RIPA lysis buffer (#P0013B, Beyotime Biotechnology) for 20 min, followed by centrifugation at 12000 rpm for 10 min at 4°C to collect the supernatant. Protein concentrations were determined using the BCA protein assay kit (#P0012, Beyotime Biotechnolog). The samples were mixed with loading buffer, boiled at 95 °C for 5 min, and stored at -20 °C until use. Equal amounts of protein were separated by SDS-PAGE and transferred onto PVDF membranes. The membranes were blocked with 5% skimmed milk at room temperature and then incubated overnight at 4 °C with primary antibodies specific for NOX4, TNF-α, PI3K, p-PI3K, AKT, p-AKT, and GAPDH. The following day, membranes were washed and incubated with horseradish peroxidase (HRP)-conjugated secondary antibody for 2 h at room temperature. After washing, the protein bands were visualized using an enhanced chemiluminescence (ECL) detection system, and images were captured using a chemiluminescence imaging system.

### Evaluation of anti-fatigue effects of MOL using juvenile zebrafish fatigue model

#### Zebrafish embryo acquisition.

Male and female zebrafish were housed separately under a 14 h light/10 h dark cycle and fed with an artificial granular diet along with newly hatched Artemia nauplii. The healthy mature zebrafish were selected and placed in a breeding tank with a 1:1 male-to-female ratio. The fertilized eggs were collected 9-10 h post-fertilization (hpf), disinfected, and washed. The eggs were then transferred to an aqueous zebrafish embryo culture medium (5.0 mM NaCl, 0.17 mM KCl, 0.4 mM CaCl_2_, and 0.16 mM MgSO_4_) and incubated at 29 °C under controlled lighting conditions.

#### Determination of LD_50_ of MOL and ROL in juvenile zebrafish.

Juvenile zebrafish at 5 days post-fertilization (dpf) were selected under stereomicroscopy and transferred to 6-well culture plates, with 15 larvae per well. MOL and the positive control Rhodiola Oral Liquid (ROL, No. B20050055, Hangzhou Huawei Pharmaceutical Co., Ltd.) were added to the culture medium at concentrations ranging from 10 to 160 μL/mL. The total volume in each well was adjusted to 6 mL with culture water. Then plates were then incubated at 29 °C in a light-controlled environment. The survival of the zebrafish was monitored 24 h after drug administration, and the LD_50_ was calculated to determine the appropriate dosage of MOL and ROL for subsequent experiments. ROL was used as the positive control due to its recognized anti-fatigue effects in vivo studies [17].

#### Effect of MOL on survival rate of fatigue zebrafish.

At 5 dpf, juvenile zebrafish were selected and transferred to 6-well culture plates with 15 fish per well. The experiment included a blank control group (aqueous embryo culture medium), a positive control group (10 μL/mL ROL), and three MOL-treated groups (5, 10, and 20 μL/mL, corresponding to MOL-L, MOL-M group, MOL-H group, respectively). The total volume in each well was adjusted to 6 mL with the culture medium. After 24 h of drug treatment, fatigue was induced in all groups except the blank control group by adding 8 mg/mL sodium sulfite (#239321, Sigma-Aldrich). The survival rate of juvenile zebrafish was observed 1 h after sodium sulfite exposure.

#### Effect of MOL on the behavior of fatigue zebrafish.

At 5 dpf, juvenile zebrafish were selected and transferred to 6-well culture plates with 15 juvenile zebrafish in each well. The experimental setup included a blank control group (embryo culture water), a positive control group (10 µ L/mL ROL), and three MOL-treated groups (5, 10, 20 µ L/mL, corresponding to MOL-L, MOL-M, and MOL-H). After 24 h of drug treatment, 6 fish from each group were transferred to 48-well plate (1 mL embryo culture water per well) for behavior analysis. In the fatigue-induced groups, 8 mg/mL sodium sulfite was used for fatigue modeling, and the behavior of the juvenile zebrafish was analyzed using a fish behavior analysis system to record movement tracks and total distance traveled within 30 min.

#### Effect of MOL on mRNA expression levels of inflammatory markers and PI3K/AKT signaling-related genes in fatigue zebrafish.

At 5 dpf, juvenile zebrafish were selected and transferred to 6-well culture plates with 15 fish per well. The experimental groups included a blank control group (aqueous embryo culture medium), a positive control group (10 μL/mL ROL), and two MOL-treated groups (10 and 20 μL/mL, corresponding to MOL-M group and MOL-H). Twenty juvenile zebrafish from each group were transferred to 1.5 mL enzyme-free sterilized EP tube for RNA extraction. RNA were extracted using the SPARKeasy tissue/cell RNA rapid extraction kit (#AC0201, SparkJade) and reverse transcribed into cDNA using NovoStart SYBR qPCR SuperMix Plus (#E096, Novoprotein). The PCR reactions were set up using HiScript III RT SuperMix for qPCR (#R323, Vazyme), with β-actin as the internal reference. The PCR conditions were as follows: predenaturation at 95°C for 60 s, followed by 40 cycles of denaturation at 95°C for 30 s, annealing at 60 °C for 30 s, and extension at 72 °C for 30 s. The relative expression levels of target genes were calculated using the 2^-ΔΔCt^. The primers sequences used are listed in the Table 3.

**Table 3.** Primer sequence information.

Gene name	Primer orientation	Nucleotide Sequence
*β-actin*	Forward	*5ʹ-AGATCGCCAAGTACTTCCAGG-3ʹ*
	Reverse	*5ʹ-GATCTGCTCTTCAATGTTGCCG-3ʹ*
*TNF-α*	Forward	*5ʹ-GGAGAGTTGCCTTTACCGCT-3ʹ*
	Reverse	*5ʹ-CCTGGGTCTTATGGAGCGTG-3ʹ*
*TNF-β*	*Forward*	*5ʹ-GGTGTCGGGGGAGTTTATCA-3ʹ*
	*Reverse*	*5ʹ-AAAAATGCAGCCACAACGCA-3ʹ*
*IL-1β*	Forward	*5ʹ-GTACTCAAGGAGATCAGCGG-3ʹ*
	Reverse	*5ʹ-CTCGGTGTCTTTCCTGTCCA-3ʹ*
*IL-6*	Forward	*5ʹ-CCATCCGCTCAGAAAACAGTG-3ʹ*
	Reverse	*5ʹ-GTTCCCCCATACTGCTGAACA-3ʹ*
*AKT1*	Forward	*5ʹ-TTTCTGCGGGATTTCAGCGG-3ʹ*
	Reverse	*5ʹ-GTCTTCACACGGGTCACCAGG-3ʹ*
*PI3K*	Forward	*5ʹ-GCAGATGGACCTTCAGATG-3ʹ*
	Reverse	*5ʹ-ATAACAGGGGGGATGACAG-3ʹ*

### Study on the material basis of MOL’s anti-fatigue effects

#### UPLC-Q-TOF/MS analysis.

MOL components were isolated and characterized using an UPLC-Q-TOF/MS system equipped with a Waters CORTECS T3 column (2.1 ×  150 mm, 1.6 μm). The mobile phase consisted of eluent A (0.1% formic acid in water, v/v) and eluent B (acetonitrile), with the followin gradient: 0-20 min, 20% B; 20-40 min, 20-50% B; 40-45 min, 50-95% B; and 45-50 min, 95% B. The flow rate was maintained at 0.25 mL/min, and the column temperature was set to 30 °C.

The UPLC system was coupled to an Agilent 6540 Q-TOF mass spectrometer equipped with an electrospray ionization source (ESI) source. Data were acquired in both negative and positive ionization modes. The ion source was operated at a capillary voltage of 3500 V, with a fragmentor voltage of 120 V. The nebulizer pressure was set to 35 psi, and high-purity nitrogen was used as both the desolvation and nebulizing gas at 300 °C. The full scan range was m/z 100-1000, and the MS/MS scan range was m/z 40-1000 with collision energies of 10, 20, and 40 eV. Data acquisition and processing were performed using MassHunter Workstation Software.

#### Serum samples preparation for Pharmacochemistry.

Mice were housed at 20 ±  2 °C under a 12 h-light/dark cycle with free access to food and water for 5 days. Six mice were randomly divided into two groups (n =  6 per group): a control group and an MOL-treated group. The MOL group received 10.8 g/kg/day of MOL, while the control group received an equivalent volume of water. Blood samples were collected from orbital sinus at 2 and 4 h post-administration. Serum was separated by centrifugation at 3500 rpm for 15 min at 25°C. The samples were pooled, treated with ACN at a ratio of 1:2, vortexed, and centrifuged at 12,000 rpm for 10 min at 4°C to precipitate proteins. The supernatant was evaporated to dryness under nitrogen at room temperature, reconstituted in 50 μL of 50% methanol-water (1:1, v/v), and subjected to UPLC-Q-TOF/MS analysis.

#### Identification strategy.

A chemical database for MOL was constructed using information from the HERB Database (http://herb.ac.cn) and relevant literature, including compound names, molecular weights, structural formulas, and MS/MS spectra. Compounds were identified by comparing experimental data with this database using MassHunter Qualitative Analysis software (Agilent Technologies, USA). Peaks with mass errors within 5 ppm were considered potential candidates. Non-matched peaks were assigned chemical formulas based on accurate mass and isotopic distributions and further identified by comparing their fragmentation patterns with those in the literature and databases such as Agilent-NatureStandard TCM PCDL, MassBank, and PubChem. Some compounds were confirmed using reference standards.

### Statistical analysis

All statistical analyses were performed using SPSS 25.0. Data were expressed as mean ±  SD ($\bar{x}\pm s$). Group comparisons were made using one-way ANOVA, and significance was determined at *P* <  0.05. Graphs were generated using GraphPad Prism 8.0 software.

## Results and discussion

### Anti-fatigue efficacy and mechanism of MOL in fatigued mice

#### Effects of MOL on body weight and organ index of mice.

After 14 days of administration, there were no significant differences in body weight or major organ indeces between the MOL-treated groups and the control group (Tables 4 and 5). These findings indicated that MOL was safe for use in mice, as there were no adverse effects on body weight or organ indices.

**Table 4:** Effect of MOL on the body weight of fatigued mice ($\bar{x}\pm s$n =  10).

Group	0d (g) (*P* value)	3d (g) (*P* value)	7d (g) (*P* value)	10d (g) (*P* value)	14d (g) (*P* value)
Control	21.7 ± 1.6	26.7 ± 1.9	30.3 ± 2.3	33.1 ± 3.2	35.2 ± 3.3
Model	21.9 ± 1.6 (0.9982)	26.1 ± 1.9 (0.9327)	29.2 ± 2.4 (0.8023)	31.2 ± 3.2 (0.6214)	33.2 ± 3.0 (0.5634)
MOL-L	22.1 ± 1.6 (0.9982)	26.5 ± 1.9 (0.9843)	29.0 ± 2.7 (0.9996)	32.0 ± 3.3 (0.9750)	34.0 ± 3.3 (0.9739)
MOL-M	21.5 ± 1.3 (0.9753)	25.9 ± 1.4 (0.9989)	28.7 ± 1.4 (0.9867)	30.9 ± 2.3 (0.9994)	32.6 ± 2.2 (0.9911)
MOL-H	21.0 ± 1.4 (0.6700)	24.4 ± 1.3 (0.1862)	26.4 ± 2.1 (0.0531)	27.9 ± 2.9 (0.1189)	29.8 ± 2.9 (0.0951)

**Table 5:** Effect of MOL on the organ index of fatigued mice ($\bar{x}\pm s$n = 10).

Group	Heart index (*P* value)	Liver index (*P* value)	Spleen index (*P* value)	Lung index (*P* value)	Kidney index (*P* value)
Control	8.91 ± 1.28	2.82 ± 0.51	1.44 ± 0.31	11.95 ± 1.65	5.11 ± 1.16
Model	9.05 ± 1.03 (0.9972)	2.96 ± 0.42 (0.9694)	1.30 ± 0.30 (0.8743)	11.57 ± 2.54 (0.9940)	5.97 ± 1.37 (0.4055)
MOL-L	9.68 ± 1.63 (0.9150)	2.94 ± 0.57 (>0.9999)	1.44 ± 0.28 (0.8743)	11.85 ± 2.68 (0.9982)	5.28 ± 0.88 (0.6202)
MOL-M	9.84 ± 2.01 (0.8259)	3.02 ± 0.40 (0.9988)	1.30 ± 0.30 (>0.9999)	12.68 ± 1.50 (0.7579)	5.91 ± 0.78 (>0.9999)
MOL-H	9.18 ± 1.90 (0.9998)	3.15 ± 0.56 (0.9116)	1.63 ± 0.43 (0.1817)	12.66 ± 1.79 (0.7698)	7.09 ± 1.15 (0.1638)

#### Effect of MOL on swimming time of mice.

As shown in Table 6, MOL significantly prolonged the weight-bearing swimming time in fatigued mice, with the highest dose (MOL-H group) showing a statistically significant difference (*P* < 0.01). The anti-fatigue ability enhancement rates for the MOL-L, MOL-M, and MOL-H groups were 11.9%, 16.9%, and 60.5%, respectively, indicating that MOL effectively improved the anti-fatigue capacity of fatigued model mice.

**Table 6:** Anti-fatigue effect of MOL in mice ($\bar{x}\pm s$n = 10).

Group	Dose	Number of animals		Swimming time (seconds)	Anti-fatigue ability Enhancement rate (%)
		Beginning	end		
Control	--	10	10	--	--
Model	--	10	10	275.8 ± 64.4	--
MOL-L	1.67mL/kg	10	10	298.4 ± 171.9	11.9%
MOL-M	3.34mL/kg	10	10	322.5 ± 103.2	16.9%
MOL-H	6.68mL/kg	10	10	442.6 ± 125.7^**^	60.5%

Note:

** *P* <  0.01 *vs.* Model group.

### Effect of MOL on liver glycogen, muscle glycogen, blood lactic acid, and blood urea nitrogen in fatigued mice

**Fig 1:**
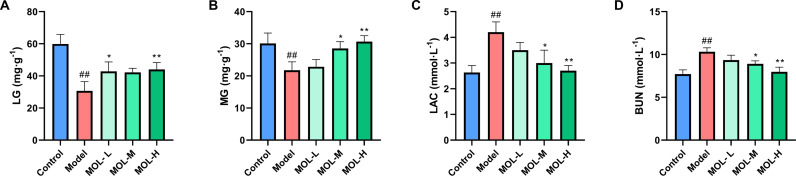
Effects of MOL on LG (A), MG (B), LAC (C), and BUN (D) in fatigued mice. Data are presented as mean ±  SD (n =  10). ^##^*P* <  0.01 *vs.* Control group; ^* ^*P* <  0.05, ^**^P <  0. 01 *vs.* Model group.

MOL showed a significant regulatory effect on liver glycogen (LG), muscle glycogen (MG), blood lactic acid (LAC), and blood urea nitrogen (BUN) level in fatigued mice (Fig 1). Post-experiment, LG and MG levels were significantly reduced in the model group compared to controls, while LAC and BUN levels increased (*P* <  0.01). MOL administration significantly improved these indices, particularly in the MOL-H group, which demonstrated a notable increase in LG and MG levels and a decrease in LAC and BUN levels compared to the model group (*P* <  0.05 or *P* <  0.01).

#### Effect of MOL on inflammatory markers in fatigued mice.

**Fig 2:**
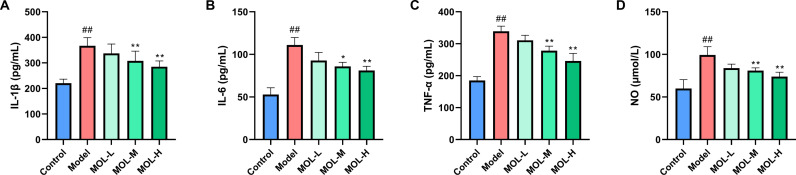
Effects of MOL on IL-1β (A), IL-6 (B), TNF-α (C), and NO (D) in fatigued mice. Data are presented as mean ±  SD (n =  10). ^##^*P* < 0.01 *vs.* Control group; ^* ^*P* <  0. 05, ^**^P < 0. 01 *vs.* Model group.

As shown in Fig 2, the levels of IL-1β, IL-6, TNF-α, and NO were significantly elevated in fatigued mice following the swimming experiment compared to controls (*P* <  0.01). MOL administration effectively reduced these levels. The MOL-M and MOL-H groups demonstrated significant reductions in IL-1β, IL-6, TNF-α, and NO levels compared to the model group (*P* <  0.05 or *P* <  0.01).

#### Effect of MOL on PI3K/AKT signaling in fatigued mice.

**Fig 3:**
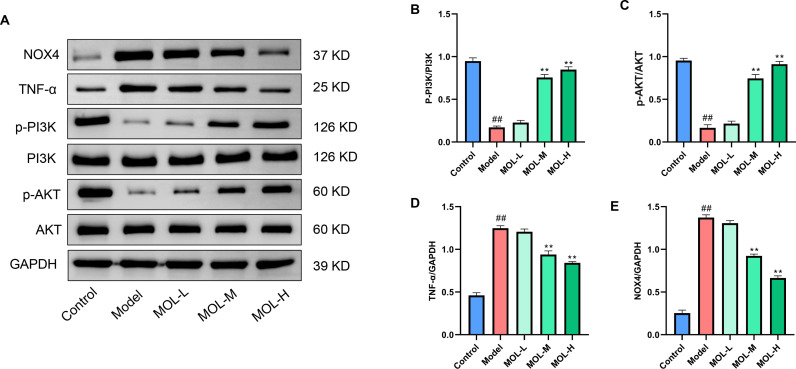
Effects of MOL on the PI3K/AKT signaling pathway in fatigued mice. (A) Representative Western blot images of NOX4, TNF-α, p-PI3K, PI3K, p-AKT, AKT, and GAPDH in the liver tissues; (B-E) Quantitative analysis of the protein expression levels of p-PI3K/PI3K (B), p-AKT/AKT (C), TNF-α (D), and NOX4 (E). Data are presented as mean ±  SD (n =  3). ^##^*P* < 0.01 *vs.* Control group; ^**^P <  0.01 *vs.* Model group.

Fig 3 illustrated the effects of MOL on key proteins involved in the PI3K/AKT signaling pathway in fatigued mice. MOL administration significantly inhibited NOX4 and TNF-α protein expression (*P* <  0.01) while increasing p-PI3K/PI3K and p-AKT/AKT levels (*P* <  0.01). The findings suggested that the anti-fatigue mechanism of MOL might involve the activation of the PI3K/AKT signaling pathway and the regulation of TNF-α and NOX4 expression in mice.

### Anti-fatigue effects of MOL in normal and fatigued zebrafish

#### Determination of MOL and ROL dosages.

**Fig 4:**
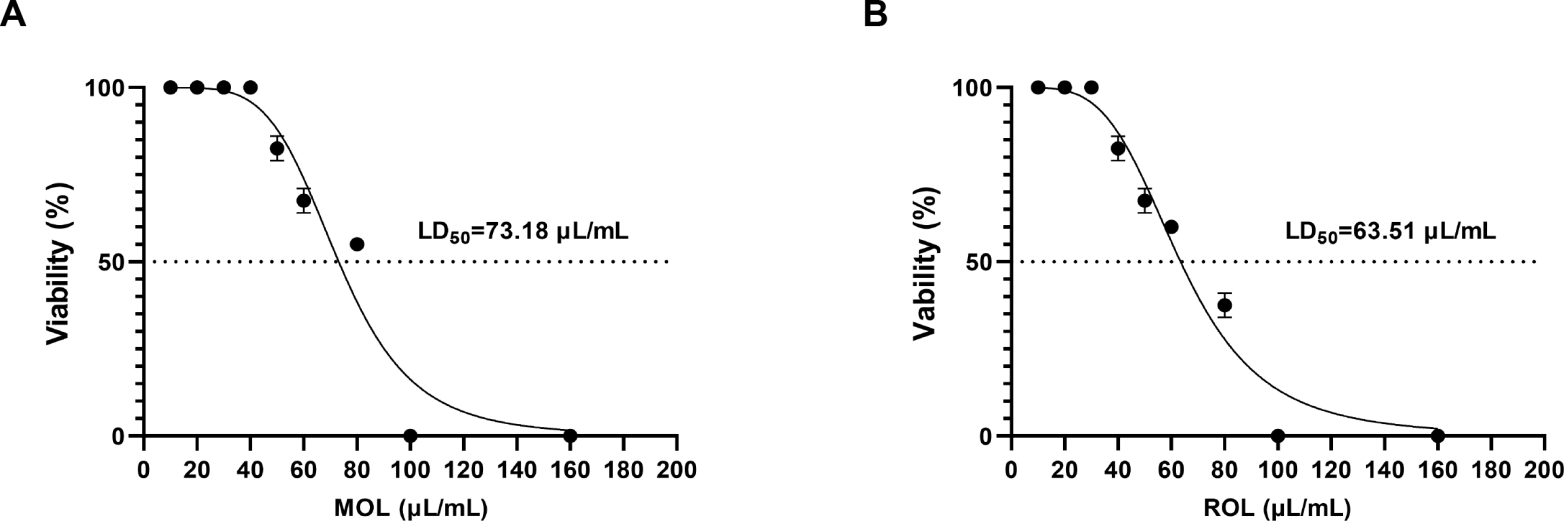
LD_50_ curve for MOL (A) and ROL (B) in juvenile zebrafish.

Due to the lack of established conversion formulas for human dosages of MOL and ROL to juvenile zebrafish, the LD_50_ of both substances was determined. As shown in Fig 4, the LD_50_ for MOL was 73.18 μL/mL, and no juvenile zebrafish died at doses below 40 μL/mL. Consequently, the experimental doses of MOL on zebrafish were set at 5, 10, and 20 μL/mL. Additionally, the LD_50_ for ROL was 63.51 μL/mL, with no fatalities at doses below 30 μL/mL. Therefore, the experimental dose of ROL on zebrafish was set at 10 μL/mL.

#### Anti-fatigue effects of MOL in normal zebrafish.

**Fig 5:**
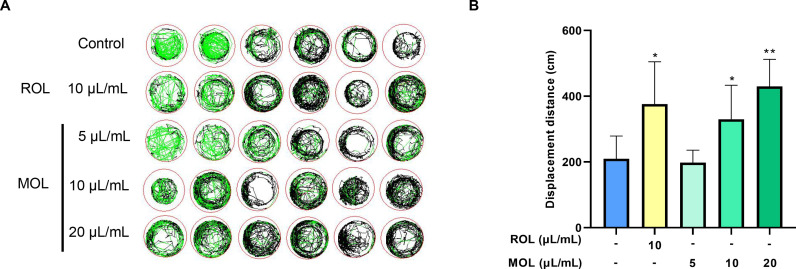
Effect of MOL on movement trajectory and distance of normal juvenile zebrafish. (A) Representative 20 min total behavior track curves of zebrafish, where the red track represents high-speed ( > 5 cm/s) motion trajectory, the black trajectory represents moderate-speed (2-5 cm/s) motion, and the green trajectory represents low-speed ( < 2 cm/s) motion path; (B) Displacement distance (cm) of zebrafish after treatment with ROL and MOL. Data are presented as mean ±  SD (n =  6). *P* <  0.05, ^**^P <  0.01 *vs.* Control group.

Fig 5 indicated that after 24 h of MOL administration, juvenile zebrafish exhibited significantly enhanced exercise abilities, as evidenced by improved high-speed motion trajectory and increased movement distance compared to the Control group. The MOL-M and MOL-H groups showed statistically significant improvements (*P* <  0.05), indicating that MOL effectively improved the exercise capacity of normal zebrafish.

### Anti-fatigue effects of MOL in fatigued zebrafish

#### Effects of MOL on survival in fatigued zebrafish.

**Fig 6:**
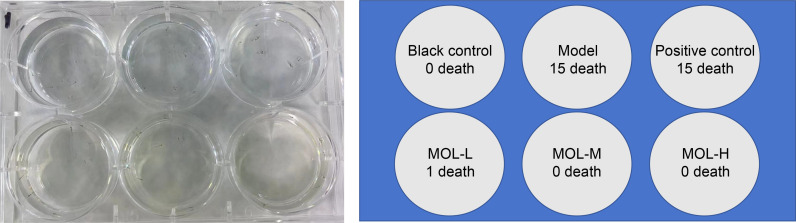
Survival of fatigue-modeled zebrafish exposed to sodium sulfite for 1 h after 24 h MOL treatment.

As shown in Fig 6, MOL significantly improved survival rates in fatigued juvenile zebrafish exposed to 8 mg/mL sodium sulfite. In the model and positive control (ROL) groups, the survival rate was 0%, indicating that ROL did not provide protection against sodium sulfite-induced mortality. However, the survival rate increased to 93.3% in the low-dose MOL group and reached 100% in both the medium and high-dose groups, demonstrating MOL’s effective protective capacity against this fatigue model.

#### Effects of ROL on motor behavior and movement distance of fatigued zebrafish.

**Fig 7:**
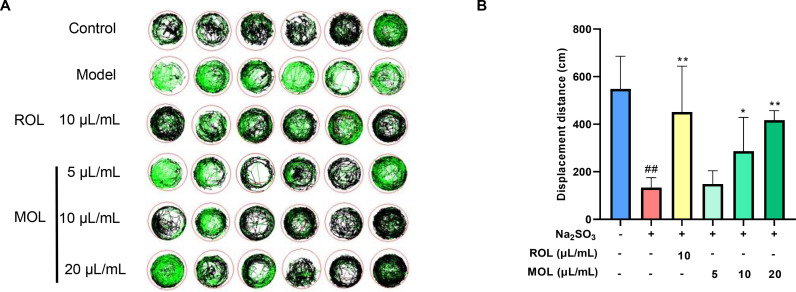
Effect of MOL on movement trajectory and distance in juvenile zebrafish fatigue model. (A) Representative 20 min total behavior track curves of zebrafish, where the red track represents high-speed ( > 5 cm/s) motion trajectory, the black trajectory represents moderate speed (2-5 cm/s) motion trajectory, and the green trajectory represents low speed ( < 2 cm/s) motion path; (B) Displacement distance (cm) of fatigued zebrafish after treatment with ROL and MOL. Data are presented as mean ±  SD (n =  6). ^##^*P* <  0.01 *vs.* Control group; ^* ^*P* <  0.05, ^**^P <  0.01 *vs.* Model group.

As shown in Fig 7, MOL significantly improved the high-speed motion trajectory and movement distance in the juvenile zebrafish fatigue model. The fatigued zebrafish in the model group exhibited a significant reduction in displacement, indicating pronounced fatigue. However, treatment with MOL, especially at medium and high doses, resulted in a substantial recovery of movement capacity (*P* <  0.05), with the highest dose nearly bringing the movement levels back to those of the control group. This improvement in motor behavior was comparable to that seen with ROL, suggesting that MOL exerted a strong anti-fatigue effect, effectively enhancing the physical performance of fatigued zebrafish.

#### Effects of MOL on mRNA expression levels inflammatory markers and PI3K/AKT signaling in fatigued zebrafish.

**Fig 8:**
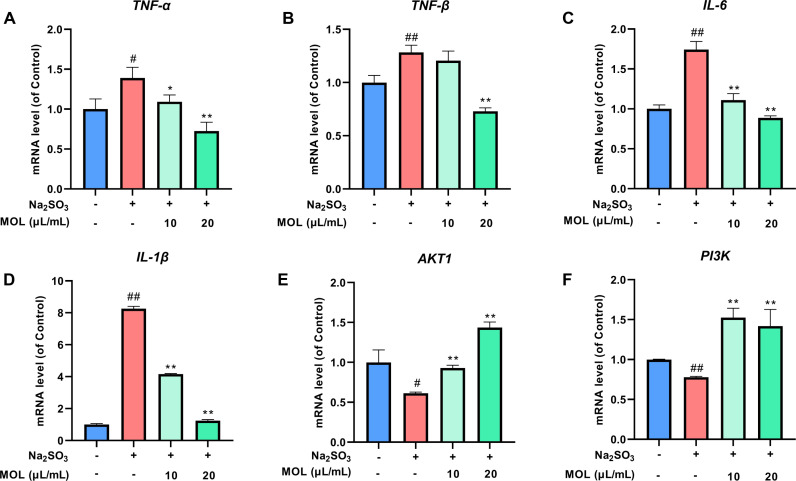
Effect of MOL on mRNA expression levels of inflammatory markers and PI3K/AKT signaling pathway-related genes in fatigued zebrafish. (A) TNF-α level; (B) TNF-β level; (C) IL-6 level; (D) IL-1β level; (E) AKT1 level; (F) PI3K level. Data are presented as mean ±  SD (n =  3). ^#^*P* <  0.05, ^##^*P* <  0.01 *vs.* Control group; ^* ^*P* <  0.05, ^**^P <  0.01 *vs.* Model group.

The mRNA expression levels of inflammatory markers (TNF-α, TNF-β, IL-6 and IL-1β) were significantly elevated in the sodium sulfite-induced fatigue model group compared to the control group, while the mRNA expression of PI3K and AKT1 was notably reduced (*P* <  0.05, Fig 8). Following 24 h of MOL treatment, there was a significant reduction in the mRNA expression levels of inflammatory markers (*P* <  0.05). Additionally, MOL treatment resulted in a significant increase in PI3K and AKT1 mRNA levels in the fatigued zebrafish (*P* <  0.01). These findings suggested that MOL exerted anti-inflammatory effects and modulated the PI3K/AKT signaling pathway in the fatigued zebrafish model.

### Chemical profiling of MOL and identification of bioactive components

**Fig 9:**
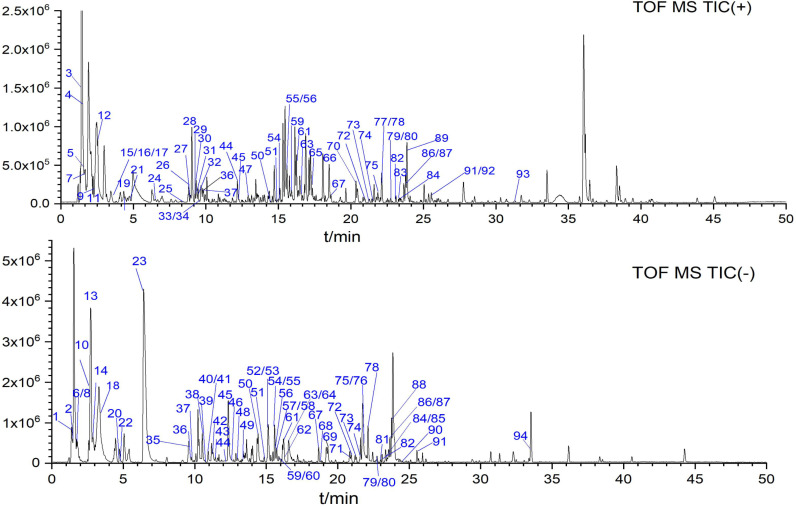
Total ion chromatograms of MOL in positive (top) and negative (bottom) ion modes using UPLC/Q-TOF MS.

The chemical composition of MOL was comprehensively analyzed using an UPLC-Q-TOF/MS method. The total ion chromatograms of MOL in positive and negative ion modes were presented in Fig 9. A total of 94 compounds were successfully identified in MOL, including 8 alkaloids, 6 amino acids, 15 flavonoids, 20 organic acids, 12 phenols, 18 phenylpropanoids, 5 nucleotides, and 11 other substances (Table 7). Identification was based on accurate mass measurements, retention times, fragmentation patterns, reference standards, and supporting literature. Among them, 19 compounds were definitively identified by direct comparison with reference standards.

**Table 7:** Identified and tentatively characterized serum components in mice treated with MOL using UPLC/Q-TOF MS.

No.	t_R_(min)		*m/z*	Mode		Formula	Identification	MS fragments (*m/z*)	Types
1	1.4		195.0514	[M-H]^–^		C_6_H_12_O_7_	Gluconic acid(^a^)	129.0205, 75.01, 59.0151	Other
2	1.4		179.0562	[M-H]^–^		C_6_H_12_O_6_	Fructose(^a^)	161.0472, 85.0306, 59.0153	Other
3	1.5		118.0867	[M+H]+		C_5_H_11_NO_2_	Betaine (^a^)	59.0733, 58.0656	Alkaloid
4	1.5		160.0969	[M+H]+		C_7_H_13_NO_3_	Medicanine(^b^)	114.091, 80.0496	Alkaloid
5	1.6		116.0708	[M+H]+		C_5_H_9_NO_2_	Proline(^b^)	70.0656, 53.0383	Amino acid
6	1.7		341.1100	[M-H]^–^		C_12_H_22_O_11_	Sucrose(^a^)	179.0582, 113.0254, 89.0259, 59.0148	Other
7#	1.8		317.1452	[M+H]+		C_12_H_20_N_4_O_6_	Pro-Ser-Asn(^a^)	202.0818, 116.0709, 70.065	Amino acid
8	1.8		133.0146	[M-H]^–^		C_4_H_6_O_5_	Malic acid(^a^)	115.0051, 71.015	Organic acid
9	1.8		144.1013	[M+H]+		C_7_H_13_NO_2_	Stachydrine(^a^)	84.0808, 58.0656	Alkaloid
10#	1.9		173.0084	[M-H]^–^		C_6_H_6_O_6_	Aconitic acid(^b^)	111.0460,93.0357,73.0293	Organic acid
11	2.3		177.0392	[M+H]+		C_6_H_8_O_6_	Vitamin C [18]	129.0138, 113.0202, 95.0095, 85.0265, 57.0323	Other
12	2.5		130.0496	[M+H]+		C_5_H_7_NO_3_	L-Pyroglutamic acid(^a^)	84.0453, 56.0504	Amino acid
13	2.7		337.0784	[M-H]^–^		C_12_H_18_O_11_	2-O-(β-d-glucopyranosyl)-ascorbic acid [19]	277.0579, 174.0184, 113.997, 59.0145	Organic acid
14	2.9		191.0186	[M-H]^–^		C_6_H_8_O_7_	Citric acid(^a^)	111.0099, 87.0101, 85.0305, 57.0357	Organic acid
15	3.3		182.0813	[M+H]+		C_9_H_11_NO_3_	Tyrosine(^a^)	96.0437, 87.0437	Amino acid
16	3.4		259.1287	[M+H]+		C_11_H_18_N_2_O_5_	g-L-Glutamyl-L-pipecolic acid(^b^)	213.1231, 170.0791, 84.0809	Organic acid
17	3.4		132.1015	[M+H]+		C_6_H_13_NO_2_	Leucine(^a^)	86.0964, 44.0498	Amino acid
18	3.9		343.0676	[M-H]^–^		C_14_H_16_O_10_	5-Galloylquinic Acid [19]	191.0588, 169.056, 125.0239, 107.0135	Organic acid
19	4.2		113.0344	[M+H]+		C_4_H_4_N_2_O_2_	Uracil(^a^)	96.0083, 70.0289	Nucleotide
20#	4.6		331.0681	[M-H]^–^		C_13_H_16_O_10_	1-Galloyl-beta-glucose [20]	271.0467, 211.0252, 169.0144, 125.0245	Phenols
21	4.8		294.1536	[M+H]+		C_12_H_23_NO_7_	Fructosyl leucine or isomer(b)	276.1454, 258.1336, 230.1383, 86.0963	Organic acid
22	4.9		331.0679	[M-H]^–^		C_13_H_16_O_10_	1-Galloyl-beta-glucose or isomer [20]	295.0412, 211.0245, 169.0145, 125.024	Phenols
23#	6.4		169.0143	[M-H]^–^		C_7_H_6_O_5_	Gallic acid(^a^)	125.0242, 79.0184, 51.0239	Organic acid
24	6.5		294.1541	[M+H]+		C_12_H_23_NO_7_	Fructosyl leucine or isomer(^b^)	276.1437, 258.1326, 230.1376	Organic acid
25	8.5		136.0617	[M+H]+		C_5_H_5_N_5_O	Adenine(^a^)	119.0327, 92.0236, 65.0135	Nucleotide
26	8.8		268.1039	[M+H]+		C_10_H_13_N_5_O_4_	Adenosine(^a^)	178.073, 136.0614, 119.0347	Nucleotide
27	8.9		194.0792	[M+H]+		C_8_H_13_NO_3_	(2S)-2-(2-Oxopyrrolidin-1-yl) butanoic acid(^b^)	164.0679, 148.078, 133.05, 108.0416, 70.028	Alkaloid
28	9.0		166.0762	[M+H]+		C_9_H_11_NO_2_	L-Phenylalanine(^a^)	120.0812, 103.0544, 77.0385	Amino acid
29	9.3		137.0460	[M+H]+		C_5_H_4_N_4_O	Hypoxanthine(^a^)	119.0337, 110.0344, 94.0387, 55.0291	Nucleotide
30	9.3		152.0569	[M+H]+		C_5_H_5_N_5_O	Hydroxytyrosol(^a^)	135.0294, 110.0345, 55.0291, 53.0131	Phenols
31	9.4		180.1020	[M+H]+		C_10_H_13_NO_2_	Daechualkaloid A [21]	133.0518, 118.0635, 77.0375, 53.0376	Alkaloid
32	9.5		330.0609	[M+H]+		C_10_H_12_N_5_O_6_P	Adenosine Cyclophosphate(^a^)	268.1138, 136.0615, 94.0641	Nucleotide
33	9.6		127.0390	[M+H]+		C_6_H_6_O_3_	5-Hydroxymethyl-2-Furaldehyde(^a^)	109.028, 87.0059, 81.0031, 53.0389	Other
34#	9.6		150.0911	[M+H]+		C_9_H_11_NO	4-Butyrylpyridine(^b^)	130.6616, 120.0506, 109.0487, 81.0575, 65.0369	Other
35	9.7		331.0674	[M-H]^–^		C_13_H_16_O_10_	1-Galloyl-beta-glucose or isomer [20]	271.0254, 211.0254, 169.0148, 125.0244	Phenols
36	9.7		483.0780	[M-H]^–^		C_20_H_20_O_14_	Gallic acid-3-O-(6’-O-galloyl)-glucoside or isomer(^b^)	331.0568, 169.0143	Organic acid
37	9.8		343.0690	[M-H]+		C_14_H_16_O_10_	Theogallin [22]	191.0568, 169.0144, 73.2345	Phenols
38	10.6		331.0678	[M-H]^–^		C1_3_H_16_O_10_	1-Galloyl-beta-glucose or isomer [20]	271.0458, 211.0245, 169.0141, 125.0238	Phenols
39	10.8		343.0675	[M-H]^–^		C_14_H_16_O_10_	4-Galloylquinic acid [23]	191.0556, 125.0245	Organic acid
40	10.9		153.0195	[M-H]^–^		C_7_H_6_O_4_	Protocatechuic acid [24]	109.0291, 108.0217	Phenols
41	10.9		109.0291	[M-H]^–^		C_6_H_6_O_2_	Catechol(^a^)	65.0035	Phenols
42#	11.3		329.0874	[M-H]^–^		C_14_H_18_O_9_	Vanillic acid glucoside [25]	165.0193, 121.0293, 109.0296, 59.0135	Organic acid
43#	11.4		315.0725	[M-H]^–^		C_13_H_16_O_9_	Maplexin B(^b^)	152.012, 108.0222, 71.0151	Phenols
44	12.2		483.0789	[M-H]^–^		C_20_H_20_O_14_	Gallic acid-3-O-(6’-O-galloyl)-glucoside or isomer(^b^)	331.0677, 169.0143	Organic acid
45	12.2		413.1915	[M+H]+		C_19_H_28_N_2_O_8_	N-(4-Aminobutyl)-3-(beta-D-glucopyranosyloxy)-4-hydroxy-trans-cinnamamide(^b^)	251.1347, 163.0387	Phenols
46	12.6		315.0725	[M-H]^–^		C_13_H_16_O_9_	Gentisic acid 5-β-glucoside or isomer(^b^)	153.0197, 109.0303, 81.0366	Other
47	12.9		188.0695	[M+H]+		C_11_H_9_NO_2_	8-Acetoxyquinoline(^b^)	146.058, 118.0625, 91.0539	Alkaloid
48	13.0		179.0344	[M-H]^–^		C_9_H_8_O_4_	Caffeic acid(^a^)	135.0445, 134.0351, 88.0296, 44.9979	Phenylpropanoid
49	13.3		353.0883	[M-H]^–^		C_16_H_18_O_9_	Neochlorogenic acid(^c^)	191.0562, 179.0347, 135.0441	Phenylpropanoid
50	14.4		483.0794	[M-H]^–^		C_20_H_20_O_14_	Gallic acid-3-O-(6’-O-galloyl)-glucoside or isomer(^b^)	331.0677, 169.0143	Organic acid
51	14.9		506.1862	[M+NH_4_]+		C_21_H_28_O_13_	Coumaroyl (-6) Fruf (b2-1a) Glc	165.0526,147.0423,85.0276	Other
52	15.0		515.1414	[M-H]^–^		C_22_H_28_O_14_	4-O-(4’-o-alpha-D-Glucopyranosyl)-caffeoyl quinic acid(^b^)	323.0771, 287.0443, 191.0555, 161.0244	Phenylpropanoid
53	15.1		483.0794	[M-H]^–^		C_20_H_20_O_14_	Gallic acid-3-O-(6’-O-galloyl)-glucoside or isomer(^b^)	313.0587, 169.0145, 125.0249	Organic acid
54#	15.1		450.1978	[M+H]+		C_19_H_28_O_11_	Zizybeoside I [26]	325.1112, 206.1331, 145.0467, 91.0526	Other
55#	15.6		344.1338	[M+NH_4_]+		C1_5_H_18_O_8_	Melilotoside [27]	165.0535, 147.0428, 119.0484, 91.0536	Phenylpropanoid
56	15.6		483.0794	[M-H]^–^		C_20_H2_0_O_14_	Gallic acid-3-O-(6’-O-galloyl)-glucoside or isomer(^b^)	313.0564,271.0481,169.0153	Organic acid
57#	15.6		163.0401	[M-H]^–^		C_9_H_8_O_3_	p-coumaric acid(^c^)	119.0505, 93.0353, 65.0405	Phenylpropanoid
58	15.7		357.0655	[M-H]^–^		C_15_H1_8_O_8_S	Tert-butyl 6-methoxy-2-methyl-5-sulfooxy-1-benzofuran-3-carboxylate or isomer(^b^)	96.9611	Other
59	15.9		355.1018	[M+H]+		C_16_H_18_O_9_	Chlorogenic acid(^c^)	193.048, 163.0366	Phenylpropanoid
60#	15.9		191.0558	[M-H]^–^		C_7_H_12_O_6_	Quinic acid(^a^)	171.0276, 127.0398, 85.03, 59.0143	Organic acid
61	16.2		353.0883	[M-H]^–^		C_16_H_18_O_9_	Cryptochlorogenic acid(^c^)	19.0561, 179.0345, 173.0449, 135.0441	Phenylpropanoid
62#	16.2		179.0336	[M+H]+		C_9_H_6_O_4_	Aesculetin(^c^)	133.0283, 123.0439, 77.0381, 51.023	Phenylpropanoid
63	16.6		293.0284	[M+H]+		C_13_H_8_O_8_	Brevifolincarboxylic Acid [28]	275.0178, 219.0276, 163.0383	Organic acid
64#	16.9		355.1031	[M-H]^–^		C_16_H_20_O_9_	1-O-feruloyl-beta-D-glucose(^b^)	193.051	Phenylpropanoid
65#	17.3		286.1432	[M+H]+		C_17_H_19_NO_3_	Coclaurine(^b^)	237.09, 175.0739, 107.048	Alkaloid
66#	18.5		342.1689	[M+H]+		C_20_H_23_NO_4_	Magnoflorine(^c^)	297.1105, 58.0644	Alkaloid
67	18.6		517.1341	[M+H]+		C_25_H_24_O_12_	Isochlorogenic acid A isomer(^b^)	337.0879, 163.0368, 79.0531	Phenylpropanoid
68#	18.9		325.0924	[M-H]^–^		C_15_H_18_O_8_	4-O-beta-Glucopyranosyl-cis-coumaric acid(^b^)	278.8557, 163.0396, 119.0501, 71.0139	Phenylpropanoid
69	19.3		163.0407	[M-H]^–^		C_9_H_8_O_3_	p-coumaric acid isomer (^a^)	119.0497, 93.0346	Phenylpropanoid
70#	20.4		193.0494	[M+H]+		C_10_H_8_O_4_	Isoscopoletin(^a^)	178.0254, 133.0283, 94.0411	Phenylpropanoid
71	20.7		473.0726	[M-H]^–^		C_22_H_18_O_12_	Chicoric aci [21]	311.0377, 278.8958, 179.0342, 161.0456	Phenylpropanoid
72	20.9	617.1142		[M+H]+	C_28_H_24_O_16_		Quercetin 3-(2-galloylglucoside) or isomer [29]	303.0467, 153.0165	Flavone
73	21.3	617.1142		[M+H]+	C_28_H_24_O_16_		Quercetin 3-(2-galloylglucoside) or isomer [29]	303.0483, 153.0185	Flavone
74	21.6	611.1613		[M+H]+	C_27_H_30_O_16_		Rutin or isomer(^a^)	465.1004, 303.0477	Flavone
75	21.8		301.0008	[M-H]^–^	C_14_H_6_O_8_		Ellagic acid(^a^)	228.0071, 200.0128, 145.0297, 117.0352	Phenols
76	21.8		339.0547	[M-H]^–^		C_15_H_16_O_9_	Esculine [21]	96.9595	Phenylpropanoid
77	21.9		465.1029	[M+H]+		C_21_H_20_O_12_	Hyperoside(^c^)	303.0491	Flavone
78#	22.1		465.1025	[M+H]+		C_21_H_20_O_12_	Isoquercitrin(^c^)	303.0493	Flavone
70#	22.8		435.0921	[M+H]+		C_20_H_18_O_11_	Guaiaverin(^b^)	303.0488, 273.0745	Flavone
80	22.8		617.1138	[M+H]+		C_28_H_24_O_16_	Quercetin 3-(2-galloylglucoside) or isomer [29]	303.0473, 153.0178	Flavone
81	23.0		515.1202	[M-H]^–^		C_25_H_24_O_12_	Isochlorogenic acid B(^a^)	431.6295, 353.0891, 179.0357, 135.0442	Phenylpropanoid
82	23.1		595.1662	[M+H]+		C_27_H_30_O_15_	Kaempferol 3-rutinoside [30]	449.1079, 287.0546	Flavone
83	23.3		598.2498	[M+H]+		C_28_ H_36_ O_13_	Acanthoside B(^c^)	401.1582, 265.1054, 205.0844	Flavone
84	23.4		611.1603	[M+H]+		C_27_H_30_O_16_	Rutin(^c^)	303.0483, 83.0484	Flavone
85	23.4		515.1201	[M-H]^–^		C_25_H_24_O_12_	Isochlorogenic acid A(^c^)	353.0886, 191.0565, 57.511	Phenylpropanoid
86#	23.7		287.0557	[M+H]+		C_15_H_10_O_6_	Demethoxycapillarisin [31]	231.0533, 165.0177, 153.018, 121.0287, 68.9972	Phenols
87#	23.7		449.1083	[M+H]+		C_21_H_20_O_11_	Homoorientin(^a^)	287.0543	Flavone
88#	23.7		461.0721	[M-H]^–^		C_21_H_18_O_12_	Luteolin-7-O-β-D-glucuronide(a)	415.2288, 346.1596, 285.0394, 229.0482	Flavone
89#	23.7		449.1078	[M+H]+		C_21_H2_0_O_11_	Astragalin(^c^)	287.0534	Flavone
90	24.4		515.1201	[M-H]^–^		C_25_H_24_O_12_	Isochlorogenic acid C(^c^)	353.0875, 191.0528	Phenylpropanoid
91	25.5		433.1132	[M+H]+		C_21_H_20_O_10_	Kaempferol-3-O-rhamnoside [32]	287.054, 129.0669, 85.027	Flavone
92	25.6		287.0553	[M+H]+		C_15_H_10_O_6_	Kaemferol(^b^)	287.0539	Flavone
93	31.4		668.4362	[M+NH_4_]+		C_36_H_58_O_10_	Kajiichigoside F1(^a^)	489.3565, 425.3397, 407.3287, 201.1642	Other
94	33.5		287.2233	[M-H]^–^		C_16_H_32_O_4_	9,10-Dihydroxy-hexadecanoic acid or isomer(^b^)	241.2162, 169.1214, 99.0814, 57.0334	Organic acid

Notes:

^a^identified by Agilent-NatureStandard Traditional Chinese Medicine Personal Compound Database and Library (TCM PCDL);

^b^identified by PubChem;

^c^identified by comparison with reference standards;

#detected in serum sample.

To further explore the bioactive constituents responsible for MOL’s anti-fatigue effects, serum samples from mice administered MOL were analyzed alongside blank serum and MOL solution. By comparing the ion peaks across these samples, 23 compounds were identified as migration constituents that were absorbed into the serum after MOL administration. These findings lay the groundwork for further studies on the active metabolites of MOL, although the focus of this study was primarily on the identification of prototype compounds rather than their metabolites.

## Discussion

Fatigue results from complex metabolic and inflammatory changes, including glycogen depletion, lactic acid accumulation, and increased levels of blood urea nitrogen (BUN), all of which serve as key biomarkers of energy consumption and physical endurance [33–35]. Inflammation further exacerbates fatigue, with elevated levels of cytokines such as TNF-α, IL-1β, and IL-6 contributing to impaired muscle function and systemic effects [36,37]. High-intensity exercise activates the iNOS/NO pathway and promotes oxidative stress, compounding fatigue symptoms through mechanisms like muscle contractility impairment [33,38–40]. These observations underscore the need for effective interventions targeting both metabolic and inflammatory pathways.

The PI3K/AKT signaling pathway plays a pivotal role in fatigue regulation by promoting glycogen synthesis and reducing inflammation [41,42]. Activation of PI3K leads to downstream phosphorylation of AKT, which subsequently modulates inflammatory proteins like TNF-α [43–45]. In addition, NADPH oxidase 4 (NOX4), a key source of reactive oxygen species (ROS), contributes to oxidative stress and fatigue progression. Regulating NOX4 expression has been shown to alleviate fatigue symptoms by mitigating oxidative damage [46]. In this study, MOL was observed to influence the PI3K/AKT pathway in both mice and zebrafish models of fatigue, albeit with some differences in the observed effects. In mice, MOL significantly increased the phosphorylation levels of PI3K and AKT, indicating an activation of the PI3K/AKT pathway, which is essential for glycogen synthesis and reducing inflammation, thus contributing to the anti-fatigue effects. In zebrafish, the study focused on the mRNA expression levels of PI3K and AKT1, rather than total AKT protein levels. AKT1 is one of the isoforms of AKT that plays a specific role in cellular survival and metabolic processes, particularly under stress conditions such as fatigue. The upregulation of PI3K and AKT1 mRNA in zebrafish after MOL treatment suggests that MOL may promote the transcription of these genes as part of its anti-fatigue mechanism. The selection of AKT1 mRNA in zebrafish over total AKT might be due to the specific involvement of AKT1 in the metabolic regulation in this model organism, where it has been implicated in responding to environmental stressors like oxidative stress and inflammation.

ROL was utilized in this study as a positive control to compare with MOL. Unlike MOL, which is a traditional medicinal formula, ROL is a well-established herbal remedy known for its adaptogenic and anti-fatigue properties, particularly in enhancing physical endurance and reducing fatigue. While ROL effectively improves motor behavior and movement distance in fatigued zebrafish, it did not significantly impact survival rates under stress conditions, as observed in this study. This difference in survival outcomes may suggest that while ROL enhances physical endurance, MOL might have additional protective effects against fatigue-induced mortality. This distinction may be attributed to MOL’s multi-compound formulation, which simultaneously targets inflammation and oxidative stress. Comparisons with other anti-fatigue agents, such as ginseng and astragalus-based formulations, further emphasize MOL’s unique advantages, including its ability to modulate the PI3K/AKT pathway and improve glycogen metabolism, distinguishing it from single-compound drugs [47,48].

In addition to these functional mechanisms, MOL’s efficacy is supported by its bioactive compounds through serum pharmacochemical analysis, which is increasingly recognized as a rational method for determining the pharmacodynamic ingredients of TCM [49,50]. Utilizing UPLC-Q-TOF/MS technology, we identified 23 migration compounds as potential active constituents. Notably, magnoflorine, a quaternary aporphine alkaloid, exhibits anti-inflammatory, neuropsychopharmacological, immunomodulatory, and antioxidant activities [51]. Luteolin-7-O-β-D-glucuronide has been shown to inhibit TNF-α mRNA expression [52]. Other identified compounds, such as aesculetin, quinic acid, and gallic acid, also demonstrate anti-inflammatory and antioxidant effects [53–55]. Additionally, astragalin, a flavonoids, activates the IL-4R/JAK1/STAT6 signaling pathway, contributing to its anti-inflammatory properties [56]. P-coumaric acid has been found to reduce TNF-α levels and ameliorate neuroinflammation in a corticosterone-induced chronic depressive mouse model [57]. Furthermore, components like luteolin-7-O-β-D-glucuronide, astragalin, 1-O-feruloyl-beta-D-glucose, and guaiaverin were found to alleviate fatigue through modulation of TNF-α [58]. This multi-component synergy may explain MOL’s comprehensive efficacy. However, further studies are needed to explore these mechanisms in greater detail.

In conclusion, MOL presented a promising therapeutic candidate for managing fatigue through its multifaceted mechanisms. However, future studies should aim to validate its effects in clinical settings and compare its efficacy with other widely recognized anti-fatigue drugs to further establish its therapeutic potential.

## Conclusion

In this study,We demonstrated that MOL exhibited a significant anti-fatigue effect. The underlying mechanism may involve the activation of PI3K/AKT signaling pathway. Notably, the inhibition of the inflammatory response appears to be a key pharmacological action through which MOL exerts its anti-fatigue effects. Using UPLC-Q-TOF/MS analysis, we identified 94 compounds in MOL. Through serum pharmacochemistry, we further identified 23 bioactive compounds that may be related to this anti-fatigue effect. Our findings provided meaningful data that could support further pharmacological validation, elucidation of the mechanism, and identification of the therapeutic material basis of MOL against fatigue. Given the limited studies on some of the potential active components, targets, and pathways identified in this research, further investigation is warranted. Additionally, molecular biology-based verification tests are necessary to confirm these findings.

## Abbreviations

AKT: protein kinase B
IL: interleukin
MOL: Meiju Oral Liquid
mRNA: messenger ribonucleic acid
NF-κB: Nuclear transcription factor
NOX4: Nicotinamide adenine dinucleotide phosphate oxidase 4
PI3K: phosphatidylinositol 3 kinase
ROL: Rhodiola oral liquid
TNF: tumor necrosis factor
UPLC-Q-TOF/MS: Ultra-performance liquid chromatography tandem time-of-flight mass spectrometry

## References

[1] Glaister M. Multiple sprint work: physiological responses, mechanisms of fatigue and the influence of aerobic fitness. Sports Med. 2005;35:757–77. doi: 10.2165/00007256-200535090-00003 16138786

[2] Nan J, Peng LJ, Wu HS, Cheng HR, Park H, Zhao QS, et al. Anti-fatigue activity and mechanism of crocetin loaded nanoliposome in acute exercise-treated mice. Food Sci Human Wellness. 2024;13:1–17.

[3] Sadeghniiat-Haghighi K, Yazdi Z. Fatigue management in the workplace. Ind Psychiatry J. 2015;24(1):12–7. doi: 10.4103/0972-6748.160915 26257477 PMC4525425

[4] Zhang X, Wang M, Zhou S. Advances in clinical research on traditional chinese medicine treatment of chronic fatigue syndrome. Evid Based Complement Alternat Med. 2020; 4715679. doi: 10.1155/2020/4715679 33343675 PMC7725552

[5] Luo C, Xu X, Wei X, Feng W, Huang H, Liu H, et al. Natural medicines for the treatment of fatigue: bioactive components, pharmacology, and mechanisms. Pharmacol Res. 2019;148:104409. doi: 10.1016/j.phrs.2019.104409 31446039

[6] Zhou SS, Jiang JG. Anti-fatigue effects of active ingredients from traditional chinese medicine: a review. Curr Med Chem. 2019;26(10):1833–48 doi: 10.2174/0929867324666170414164607 28413958

[7] Huang X, An XX, Fang JP, Li JB, Xi CF. Study on anti-fatigue and anti-oxidation of rose anthocyanins in mice. J Yunnan Agric Univ Nat Sci. 2021;36:956–61. doi: 10.12101/j.issn.1004-390X(n).202104053

[8] Fu AY, Wang Q, Wu YL, Zhou F, Wang ZJ, Wang RL, et al. 2018. In vitro antioxidant activity and anti-fatigue effect of chicory polysaccharide. Sci Technol Food Ind. 2018;39(9):1–5. doi: 10.13386/j.issn1002-0306.2018.09.001

[9] Chen YH, Zhu M, Li BL, Fu ZY, Feng G. Study of soft capsule of compound oil of jujube, arborvitae, and gardenia on enhancing hypoxia tolerance and anti-fatigue in mice. Zhongguo Ying Yong Sheng Li Xue Za Zhi. 2012;28(4):339–41. 23156731

[10] Yang CX, Yang JY, Tan L, Pen T, Gao TH, Liu SJ, et al. A novel formula comprising wolfberry, figs, white lentils, raspberries, and maca (WFWRM) induced antifatigue effects in a forced exercise mouse model. Evid Based Complement Alternat Med. 2022;3784580. doi: 10.1155/2022/3784580 35368749 PMC8970811

[11] Zhang JP, Zhang LL, Nur AN, Lai HZ, Xiao XW, Ali MAL. Preliminary study on effects of Meiju oral Liquid on fatigue resistance and hypoxia tolerance in mice. Renowned Doc. 2020;37–39.https://api6.wenxian.shop/v1/api/download?dflag=pdfdown&v=H/AbukAGNHIw21fBi4ZSVAN3tUAP3qg9bQdPMnnl8y4PBhOKD2TQpr/qLB7ytmVydmizk2mAouutJAMoHo5J/POXsHx4kolaMgpSeKE1hFdOq51kuwnWSpQjv8J6uwXlEJeXOi±THUmV4gcIQ0erNgzoJK95Y5n/fK8Kz1w6Kqk4JMdx4NjW1y7OJlV7hXBPdcTwbA3fnELLb0tXlceRnjXorEwxbzBvPlwO6X9TxPE2Cy98GbXyPN5iOUQM5H2W&fileid=MGYI202016022&dataDbname=CJFQ

[12] Zhang LL, Zainabu D, Zhang JP, Maiwulanjiang M. Study on the effect of Meiju oral liquid on exercise fatigue in mice based on nuclear transcription factor E2- related factor 2/heme oxygenase-1 signaling pathway. Chin J Clin Pharmacol. 2023;39(12):1773–77. doi: 10.13699/j.cnki.1001-6821.2023.12.021

[13] Jaime-Lara RB, Koons BC, Matura LA, Nancy A, Hodgson NA, Riegel B, et al. A qualitative metasynthesis of the experience of fatigue across five chronic conditions. J Pain Sympt Manage. 2020;59(6):1320–43. doi: 0.1016/j.jpainsymman.2019.12.358 31866485 10.1016/j.jpainsymman.2019.12.358 PMC7239763

[14] Wan JJ, Qin Z, Wang PY, Sun Y, Liu X. Muscle fatigue: general understanding and treatment. Exp Mol Med. 2017;49:e384.doi: 10.1038/emm.2017.194 28983090 PMC5668469

[15] Zhao R, Wu R, Jin J, Ning K, Wang Z, Yi X, et al. Signaling pathways regulated by natural active ingredients in the fight against exercise fatigue-a review. Front Pharmacol. 2023;14:1269878. doi: 10.3389/fphar.2023.1269878 38155906 PMC10752993

[16] Liu S, Meng F, Zhang D, Shi D, Zhou J, Guo S, et al. Lonicera caerulea berry polyphenols extract alleviates exercise fatigue in mice by reducing oxidative stress, inflammation, skeletal muscle cell apoptosis, and by increasing cell proliferation. Front Nutr. 2022;9:853225. doi: 10.3389/fnut.2022.853225 35356725 PMC8959458

[17] Hou Y, Tang Y, Wang X, Ai X, Wang H, Li X, et al. Rhodiola Crenulata ameliorates exhaustive exercise-induced fatigue in mice by suppressing mitophagy in skeletal muscle. Exp Ther Med. 2020;20(4):3161–73. doi: 10.3892/etm.2020.9072 32855685 PMC7444336

[18] Smith AG, Han E, Petersen J, Olsen NAF, Giese C, Athmann M, et al. RootPainter: deep learning segmentation of biological images with corrective annotation. New Phytol. 2022;236:774–91. doi: 10.1111/nph.18387 35851958 PMC9804377

[19] Dong W, Peng YJ, Chen GJ, Xie ZY, Xu W, Zhou WT, et al. 2-O-β-D-Glucopyranosyl-L-ascorbic acid, an ascorbic acid derivative isolated from the fruits of Lycium barbarum L., ameliorates high fructose-induced neuroinflammation in mice: involvement of gut microbiota and leaky gut. Food Sci Hum Wellness. 2024;13:241–53. https://kns.cnki.net/dm8/manage/export.html?language=CHS&uniplatform=NZKPT

[20] Chen MQ, Zhu WF, Guan YM, Feng YL, Zhang YL, Jing XC, et al. Analysis of chemical constituents in puerariae lobatae radix dispensing granules by UPLC-Q-TOF-MS/MS. Chinese J Exp Trad Med Formulae. 2023;29:176–86. doi: 10.13422/j.cnki.syfjx.20230762

[21] Zhu CS, Lin ZJ, Zhang B, Niu HJ, Wang XJ, Zhang XM, et al. Qualitative and quantitative analysis of chicory root by LC/MS and HPLC. J Beijing Univ Tradit Chin Med. 2016;39;247–51. doi: 10.3969/j.issn.1006-2157.2016.03.014

[22] Han S, Zheng W, Nan Y, et al. Aanalysis of chemical components of wuyi rock tea based on UHPLC-Q-TOF/MSE. Mod Food Sci Technol. 2016;39:247–51. doi: 10.13982/j.mfst.1673-9078.2022.6.0962

[23] Clifford MN, Stavroula S, Nikolai K. Profiling and characterization by LC-MSn of the galloylquinic acids of green tea, tara tannin, and tannic acid. J Agric Food Chem. 2007;55 (8):2797–807. doi: 10.1021/jf063533l 17381119

[24] Feng ML, Wang SF, Zhang XX. Chemical constituents in fruits of lycium barbarum. Chin Tradit Herb Drugs. 2013;44:265–68. https://kns.cnki.net/kcms2/article/abstract?v=qwZretP9BaHxjSrRsOauxCDX5ONtG8a8rPLZHbirKCiJSIXWyiVWR9WIwve5N0Pgu7CMKqpqy_ncGwaPzw4SlRImJe-p3jXgu1r6UzOa7qzeiSaYPmm63ObvU-FXoVtqeD99_hkpYYs=&uniplatform=NZKPT&language=CHS

[25] Ma BJ, Xiao Y, Chen ZD, Shu RK, Li BT, Jiang L, et al. Analysis of chemical constituents in percolate the extract of cyclocarya paliurus tender leaves by UHPLC-Q-TOF-MS/MS[J]. Sci Technol Food Industry. 2023;44(13):281–91. doi: 10.13386/j.issn1002-0306.2022070294

[26] Liu SJ, Tang ZS, Cui CL, Liu HB, Liang YN, Zhang Y, et al. Advances in Studies on Chemical Constituents of Ziziphus jujuba. J YunNan Univ Chin Med. 2015;38:96–100. doi: 10.19288/j.cnki.issn.1000-2723.2015.03.027

[27] Olennikov DN, Chirikova NK, Kashchenko NI, Nikolaev VM, Kim SW, Vennos C, et al. Bioactive phenolics of the genus artemisia (Asteraceae): HPLC-DAD-ESI-TQ-MS/MS profile of the siberian species and their inhibitory potential against α-amylase and α-glucosidase. Front pharmacol. 2018;9:756. doi: 10.3389/fphar.2018.00756 30050443 PMC6052120

[28] Tan LW, Jin HH, Liu YX, Qian Z, Shi JH, Wu D, et al. Main chemical constituents of Changyanning Tablets based on HPLC-Q-TOF-MS/MS. Chin Tradit Herb Drugs. 2020;51:4124–32. doi: 10.7501/j.issn.0253-2670

[29] Ismail S, Chandel TI, Ramakrishnan J, Khan RH, Poomani K, Devarajan N, et al. Phytochemical profiling, human insulin stability and alpha glucosidase inhibition of Gymnema latifolium leaves aqueous extract: exploring through experimental and in silico approach. Comp biol chem. 2023;107:107964. doi: 10.1016/j.compbiolchem.2023.107964 37820470

[30] Ma X. Study on the extraction process and component identification of polyphenolic compounds from lycium barbarum. Xinjiang Agricult Univ. 2022. https://kns.cnki.net/kcms2/article/abstract?v=lQz6UQjnwp_RdX0g7_iT5zCKLT1YhFKQ0fx-FvOEnkii1yDEhsvp_se-N15SZqW7hzDylPj7YWc4BkcTRZO2laeq9-4EkpyGS-vnn0yLyGeNKEEX5WhHRANpCs1QCo0TTtVtLtm

[31] Sithes L, David MR, Yang H, Alexander P, Ma J, Edward JK, et al. Bioassay-guided isolation of aldose reductase inhibitors from Artemisia dracunculus. Phytochemistry. 2006;67(14):1539–46. doi: 10.1016/j.phytochem.2006.05.015 16806328

[32] Zhang Q. Studies on the chemical constituents and biological activities of fructus sophorae and nelumbinis plumula, jilin university. 2018. https://kns.cnki.net/kcms2/article/abstract?v=lQz6UQjnwp_XvAyie6SvQ8FmsbR04a1sKhhQ-QP8ZpJ9-dwDlBWGqdZ4m-ww1dSYneR7AO8BhwAgAVhqC1um9df1HsA9Fe6WSmxzXwQu1LPjs_hqIl7xLbKg8QXFh0S-0xoLIIO0LAc=&uniplatform=NZKPT&language=CHS

[33] Klimas NG, Broderick G, Fletcher MA. Biomarkers for chronic fatigue. Brain Behav Immun. 2012;26(8):1202–10. doi: 10.1016/j.bbi.2012.06.006 22732129 PMC5373648

[34] Li A. Effects and mechanisms of jujube on intestinal inflammatory injury and feeding in T2DM rats by regulating NF-κB signaling pathway. Chengdu University of TCM. 2023. doi: 0.26988/d.cnki.gcdzu.2023.000131

[35] Zhang L, Huang CL, Chen YX. Relationship between CNTF gene polymorphism and athletes training adaptation of muscle tissue. Zhonghua Yi Xue Za Zhi. 2013;93(37):2969–71. 24401586

[36] Bi Y, Liu X, Liu Y, Wang M, Shan Y, Yin Y, et al. Molecular and biochemical investigations of the anti-fatigue effects of tea polyphenols and fruit extracts of Lycium ruthenicum Murr. on mice with exercise-induced fatigue. Front Molecul Biosci. 2023;10: 1223411. doi: 10.3389/fmolb.2023.1223411 37416624 PMC10319583

[37] Packer N, Hoffman-Goetz L. Acute exercise increases hippocampal TNF-α, Caspase-3 and Caspase-7 expression in healthy young and older mice. J Sports Med Phys Fitness. 2015;55(4):368–76. 25853879

[38] Lee BR, Lee JH, An HJ. Effects of Taraxacum officinale on fatigue and immunological parameters in mice. Molecules. 2012;17(11):13253–65. doi: 10.3390/molecules171113253 23135630 PMC6268574

[39] Liu S, Meng F, Zhang D, Shi D, Zhou J, Guo S, et al. Lonicera caerulea Berry Polyphenols Extract Alleviates Exercise Fatigue in Mice by Reducing Oxidative Stress, Inflammation, Skeletal Muscle Cell Apoptosis, and by Increasing Cell Proliferation. Frontiers Nutr. 2022;9:853225. doi: 10.3389/fnut.2022.853225 35356725 PMC8959458

[40] Kim HY, Han NR, Kim NR, Lee M, Kim J, Kim CJ, et al. Effect of fermented porcine placenta on physical fatigue in mice. Exp Biol Med (Maywood, N.J.). 2016;241(17):1985–96. doi: 10.1177/1535370216659945 27439540 PMC5068459

[41] Hu M, Han MX, Zhang H, Li ZF, Xu KY, Kang HX, et al. Curcumin (CUMINUP60®) mitigates exercise fatigue through regulating PI3K/Akt/AMPK/mTOR pathway in mice. Aging (Albany NY). 2023;15(6):2308–20. doi: 10.18632/aging.204614 Epub 2023 Mar 28. 36988546 PMC10085593

[42] Yeung YT, Aziz F, Guerrero-Castilla A, Arguelles S. Signaling pathways in inflammation and anti-inflammatory therapies. Curr Pharm Des. 2018;24(14):1449–84 doi: 10.2174/1381612824666180327165604 29589535

[43] Lei C, Chen J, Huang Z, Men Y, Qian Y, Yu M, et al. Ginsenoside Rg1 can reverse fatigue behavior in CFS rats by regulating EGFR and affecting Taurine and Mannose 6-phosphate metabolism. Front Pharmacol. 2023;14:1163638. doi: 10.3389/fphar.2023.1163638 37101547 PMC10123289

[44] Burke JE, Williams RL. Synergy in activating class I PI3Ks. Trends Biochem Sci. 2015;40(2):88–100. doi: 10.1016/j.tibs.2014.12.003 25573003

[45] Liu X, Xu Y, Zhou Q, Chen M, Zhang Y, Liang H, et al. PI3K in cancer: its structure, activation modes and role in shaping tumor microenvironment. Future oncol (London, England). 2018;14(7):665–74. doi: 10.2217/fon-2017-0588 29219001

[46] Liu S, Meng F, Zhang D, Shi D, Zhou J, Guo S, et al. Lonicera caerulea berry polyphenols extract alleviates exercise fatigue in mice by reducing oxidative stress, inflammation, skeletal muscle cell apoptosis, and by increasing cell proliferation. Front Nutr. 2022;9:853225. doi: 10.3389/fnut.2022.853225 35356725 PMC8959458

[47] Ahn S, Jamrasi P, Lim B, Seo JW, Li X, Jiang S, et al. Herbal extract (Cervus elaphus Linnaeus, Angelica gigas Nakai, and Astragalus membranaceus Bunge) ameliorates chronic fatigue: a randomized, placebo-controlled, double-blind trial. Integrat Med Res. 2024;13(4):101085. doi: 10.1016/j.imr.2024.101085 39399821 PMC11465177

[48] Lu G, Liu Z, Wang X, Wang C. Recent advances in panax ginseng C.A. meyer as a herb for anti-fatigue: an effects and mechanisms review. Foods (Basel, Switzerland) 2021;10(5):1330. doi: 10.3390/foods10051030 34068545 PMC8151278

[49] Yin FT, Zhou XH, Kang SY, Li XH, Li J, Ullah I, et al. Prediction of the mechanism of Dachengqi Decoction treating colorectal cancer based on the analysis method of “ into serum components -action target-key pathway”. J Ethnopharmacol. 2022;293:115286. doi: 10.1016/j.jep.2022.115286 35413412

[50] Shao D, Liu X, Wu J, Zhang A, Bai Y, Zhao P, et al. Identification of the active compounds and functional mechanisms of Jinshui Huanxian formula in pulmonary fibrosis by integrating serum pharmacochemistry with network pharmacology. Phytomedicine. 2022;102:154177. doi: 10.1016/j.phymed.2022.154177 35636171

[51] Xu T, Kuang T, Du H, Li Q, Feng T, Zhang Y, et al. Magnoflorine: A review of its pharmacology, pharmacokinetics and toxicity. Pharmacol Res. 2020;152:104632. doi: 10.1016/j.phrs.2020.104632 31911246

[52] Cho YC, Park J, Cho S. Anti-inflammatory and anti-oxidative effects of luteolin-7-O-glucuronide in LPS-stimulated murine macrophages through TAK1 inhibition and Nrf2 activation. Int J Mol Sci. 2020;21(6):2007. doi: 10.3390/ijms21062007 32187984 PMC7139836

[53] Wang SK, Chen TX, Wang W, Xu LL, Zhang YQ, Jin Z, et al. Aesculetin exhibited anti-inflammatory activities through inhibiting NF-кB and MAPKs pathway in vitro and in vivo. J Ethnopharmacol. 2022;296:115489. doi: 10.1016/j.jep.2022.115489 35728711

[54] Tanaka M, Kishimoto Y, Sasaki M, Sato A, Kamiya T, Kondo K, et al. Terminalia bellirica (Gaertn.) roxb. extract and gallic acid attenuate LPS-induced inflammation and oxidative stress via MAPK/NF-κB and Akt/AMPK/Nrf2 pathways. Oxid med cell longev. 2018;9364364. doi: 10.1155/2018/9364364 30533177 PMC6250009

[55] Jang SA, Park DW, Kwon JE, Song HS, Park B, Jeon H, et al. Quinic acid inhibits vascular inflammation in TNF-α-stimulated vascular smooth muscle cells. Biomed Pharmacother. 2017;96:563–71. doi: 10.1016/j.biopha.2017.10.021 29032340

[56] Yao G, Bai Z, Niu J, Zhang R, Lu Y, Gao T, et al. Astragalin attenuates depression-like behaviors and memory deficits and promotes M2 microglia polarization by regulating IL-4R/JAK1/STAT6 signaling pathway in a murine model of perimenopausal depression. Psychopharmacology. 2022;239(8):2421–43. doi: 10.1007/s00213-022-06133-5 35411464

[57] Yu XD, Zhang D, Xiao CL, Zhou Y, Li X, Wang L, et al. P-coumaric acid reverses depression-like behavior and memory deficit via inhibiting age-rage-mediated neuroinflammation. Cells. 2022;11(10):1594. doi: 10.3390/cells11101594 35626632 PMC9139330

[58] Akazawa KH, Cui Y, Tanaka M, Kataoka Y, Yoneda Y, Watanabe Y. Mapping of regional brain activation in response to fatigue-load and recovery in rats with c-Fos immunohistochemistry. Neurosci Res. 2010;66(4):372–9. doi: 10.1016/j.neures.2009.12.009 20018215

